# Supplementary material for: Super-resolution architecture of mammalian centriole distal appendages reveals distinct blade and matrix functional components
Source: Nat Commun. 2018 May 22;9:2023. doi: 10.1038/s41467-018-04469-1 (PMC5964178; doi:10.1038/s41467-018-04469-1)
Supplement: Supplementary file 1 — Supplementary Information [file 41467_2018_4469_MOESM1_ESM.pdf]

A

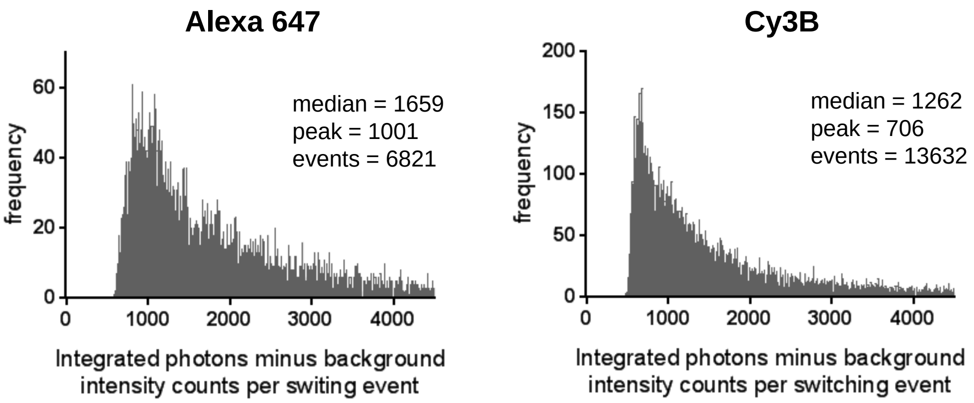

B

$$\sigma = \sqrt{\frac{s^2}{N} + \frac{a^2/12}{N} + \frac{8\pi s^4 b^2}{a^2 N^2}}$$

CCD gain = 2.4e$^-$ / ADU
EM gain = 200 for Alexa 647 and 150 for Cy3B

	Alexa 647	Cy3B
photons, *N*	1001	706
standard deviation of PSF distribution, *s* (nm)	148	134
pixel size, *a* (nm)	93	93
standard deviation of the background, *b* (photons)	5.43	6.65
effective background DC level (photons)	30.2	23.5
localization precision in FWHM (nm)	18.76	24.65

Supplementary Figure 1 Evaluation of localization precision of the dSTORM system.

(A) Measured photon distributions of Alexa 647 and Cy3B from fixed samples labeled with typical ciliary markers. The samples incubated in an imaging buffer containing 80 mM MEA were illuminated at an intensity of around 2 kW cm^-2. The number of photons detected per switching event was registered as the total integrated signal minus the average background level and then converted to photon counts using the electron multiplication gain settings and analog-to-digital conversion gain (values shown in (B)). (B) Calculation of localization precision. The localization precision (σ) of our system was estimated using the shown equation (left) and the associated parameters (right). The standard deviation (s) of a PSF was obtained through a 2D Gaussian function-fit to the PSF distribution. For the photon count (N), we used the

value at the peak occurrence frequency of the photon number distribution for each case (A). To characterize the average background DC level and the standard deviation of the background (b), intensity histograms of three image sets with noises only were analyzed and the values were converted to effective photon counts. The localization precisions in FWHM were calculated based on the equation.

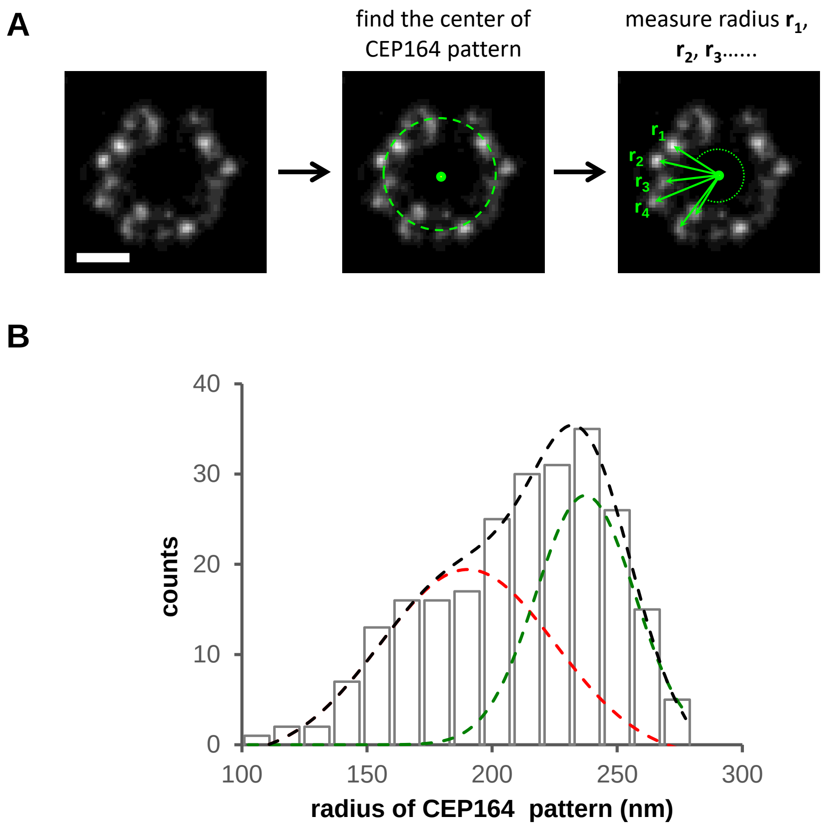

Supplementary Figure 2 Radial distribution analysis of CEP164 puncta to define inner and outer radii.

(A) The CEP164 pattern was first fitted with a circle, and the radius was then defined as the distance between the CEP164 puncta and the center of the circle. (B) The radial histogram was fitted with a mixed function to determine the inner and outer radii. Bar = 200 nm.

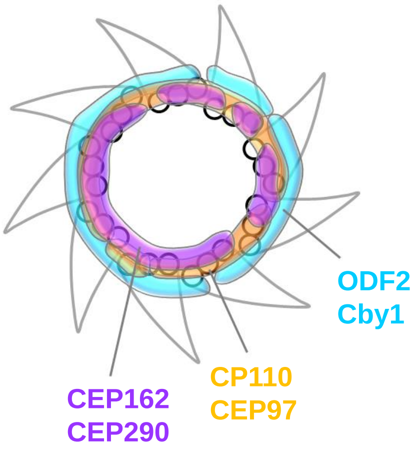

Supplementary Figure 3 Model of the localization of DAP-related proteins. CP110, CEP97, CEP290 and CEP164 are situated at a similar radial distance near the axoneme.

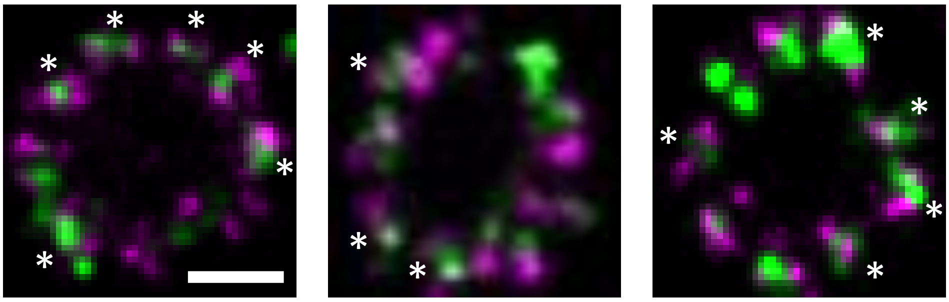

Supplementary Figure 4 Two-color dSTORM axial view images of CEP164 (magenta) and SCLT1 (green).

Asterisks indicate SCLT1 puncta lying between CEP164 signals. In three dual-color images, 14 out of 27 SCLT1 puncta localize within the ‘blades’ of CEP164 propeller-like signals. Bar = 200 nm.

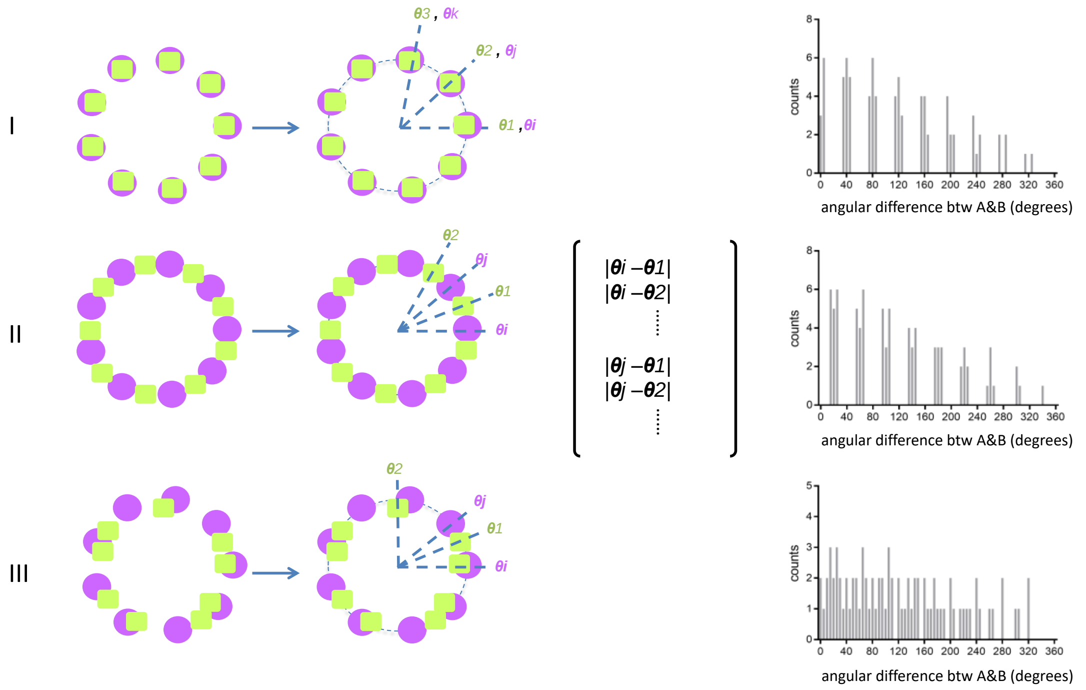

Supplementary Figure 5 The method used for angular analysis.

Angular analysis was used to determine the relative circumferential positions between each of a DAP protein pair (observed by axial view dSTORM). The angular position of each protein punctum was first recorded, and the difference in angles was then calculated by subtracting the angular position of the punctum of one protein (e.g. θ_1, θ_2…) from that of the other protein (e.g. θ_i, θ_j…). If puncta of protein A (magenta circles) and B (green circles) are both equally distributed and align well with each other (scenario I), angle differences between their puncta will be 40° (360° divided by 9) or multiples of 40°. If puncta of proteins A and B are organized in an alternating manner (scenario II), their neighboring puncta will exhibit a difference of 20° or multiples of 40° plus 20°. Finally, if the two proteins are randomly distributed (scenario III), a wide range of angular differences will be recorded.

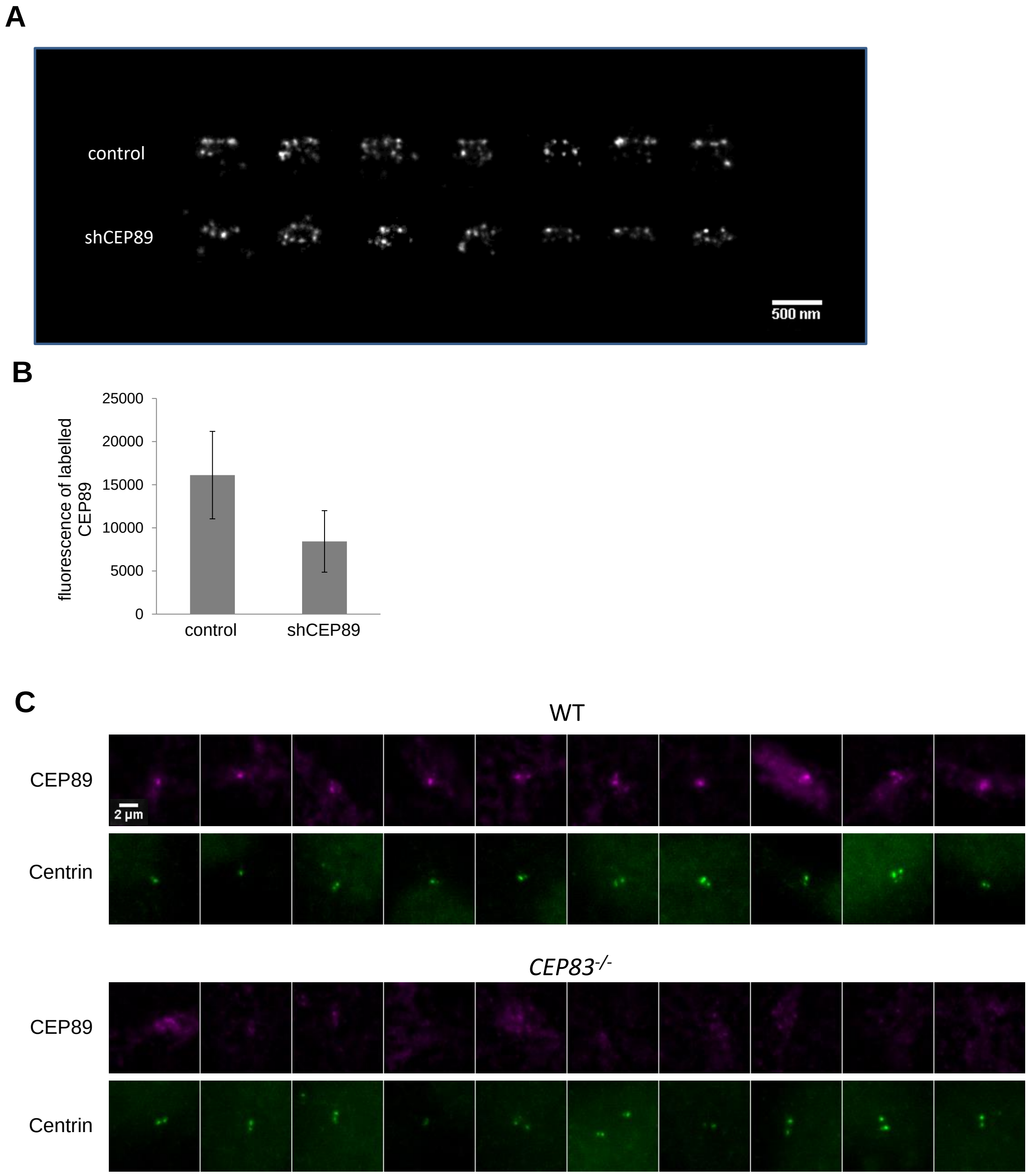

Supplementary Figure 6 Specificity test for CEP89 antibody-labeled signals.

(A) dSTORM imaging of CEP89 in shCEP89 cilia showing that both layers of CEP89 are evenly disrupted in knockdown cells, indicating that the bottom layer did not arise from nonspecific labeling. (B) CEP89 depletion by shRNA quantified by measuring the CEP89 fluorescence intensity (n = 17 for control and n = 9 for shCEP89). (C) In *CEP83*$^{-/-}$ centrioles, where CEP89 was previously reported to be absent, CEP89 signals were not observed, demonstrating the specificity of the CEP89 antibody.

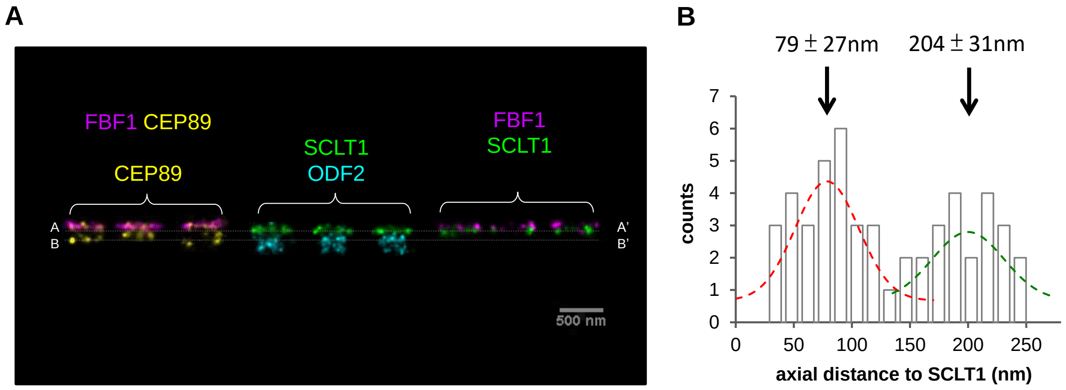

Supplementary Figure 7 Double-layered ODF2 revealed by localization microscopy. (A) Superresolved ODF2 exhibits a two-layer distribution with the distal layer localized near the proximal layer of CEP89. (B) Histogram of the longitudinal positions of ODF2 relative to SCLT1. Two peak positions were fitted with a Gaussian mixture function, yielding central positions at 79 nm and 204 nm from SCLT1. The observation of two layers of ODF2 occupancy may reflect the previous finding that ODF2 affects the formation of both DAPs and the subdistal appendage [1].

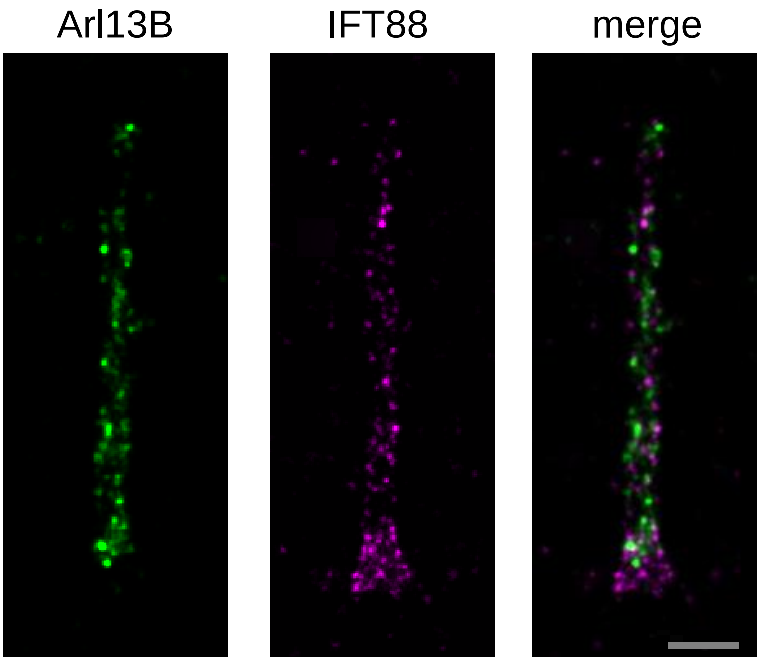

Supplementary Figure 8 ARL13B and IFT88 cover a similar width along the cilium proper. Bar = 500 nm.

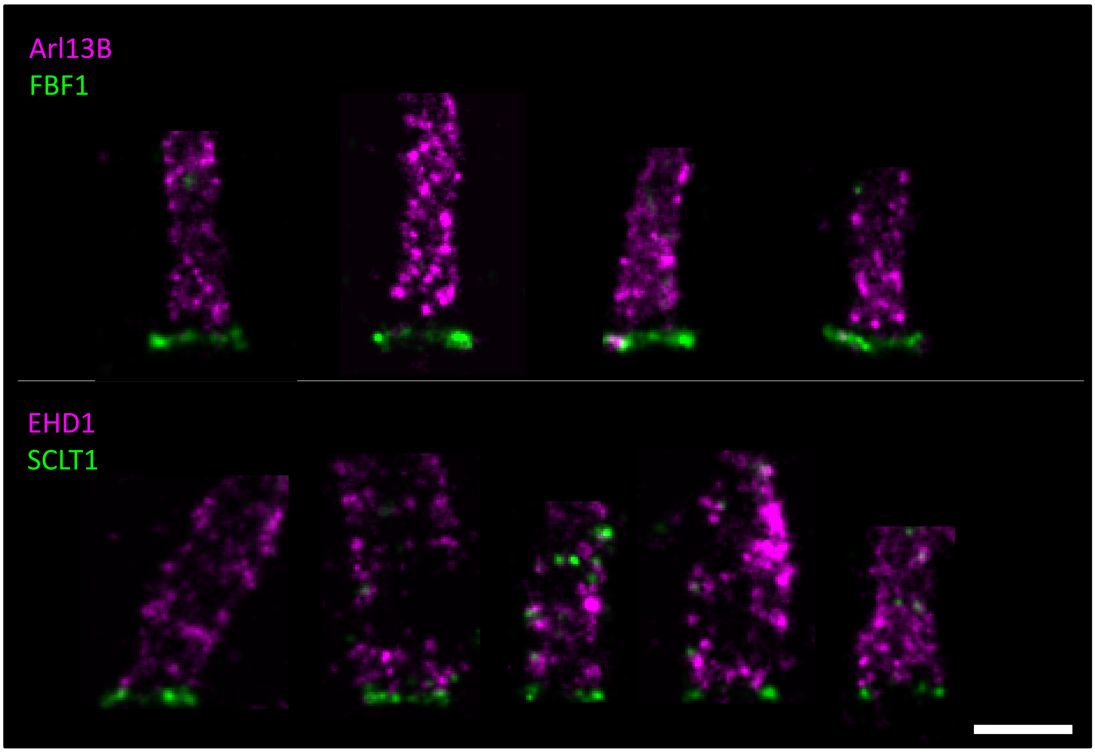

Supplementary Figure 9 Differences in the width coverage and distal appendage (DAP) proximity of ARL13B and EHD1.

The width of the region occupied by EHD1 along the cilium is wider than that occupied by ARL13B, consistent with EHD1 localization in ciliary pockets. An exclusion zone between DAPs (labeled with FBF1) and the proximal end of ARL13B is evident, whereas no gap is observed between the proximal end of EHD1 and DAPs (labeled with SCLT1). Bar = 500 nm.

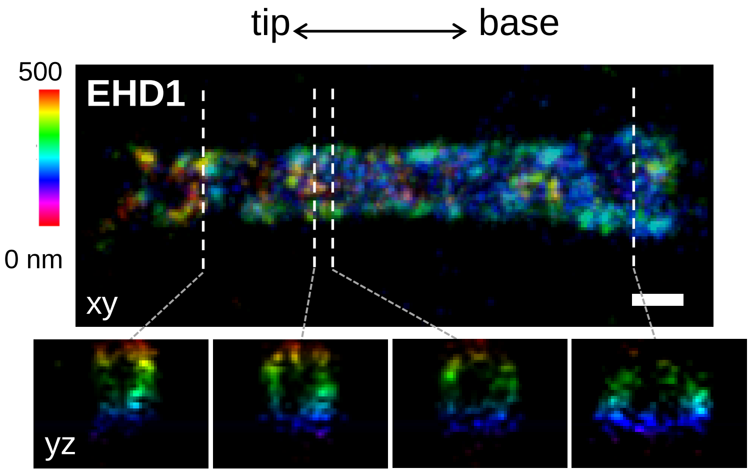

Supplementary Figure 10 A 3D dSTORM snapshot showing the peripheral distribution of EHD1 enveloping the primary cilium.

Cross-section images at four different locations (dashed lines) from DAP region toward the ciliary tip are shown in the lower panels. Bar = 200 nm.

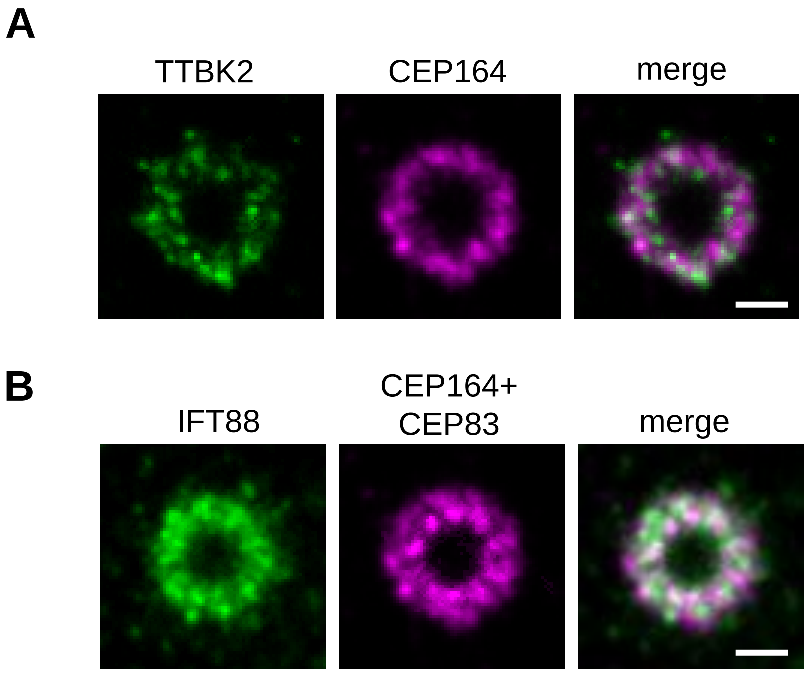

Supplementary Figure 11 Radial distribution analysis indicating distinct TTBK2 and IFT88 coverages.

(A) Overlaid TTBK2 dSTORM (n = 11) and overlaid CEP164 (n = 13) images cover a similar radial range, as illustrated in the merged panel on the right. (B) The overlaid IFT88 image (n = 13), however, covers a different range, spanning from CEP164 to CEP83 (n = 11). Bars = 200 nm.

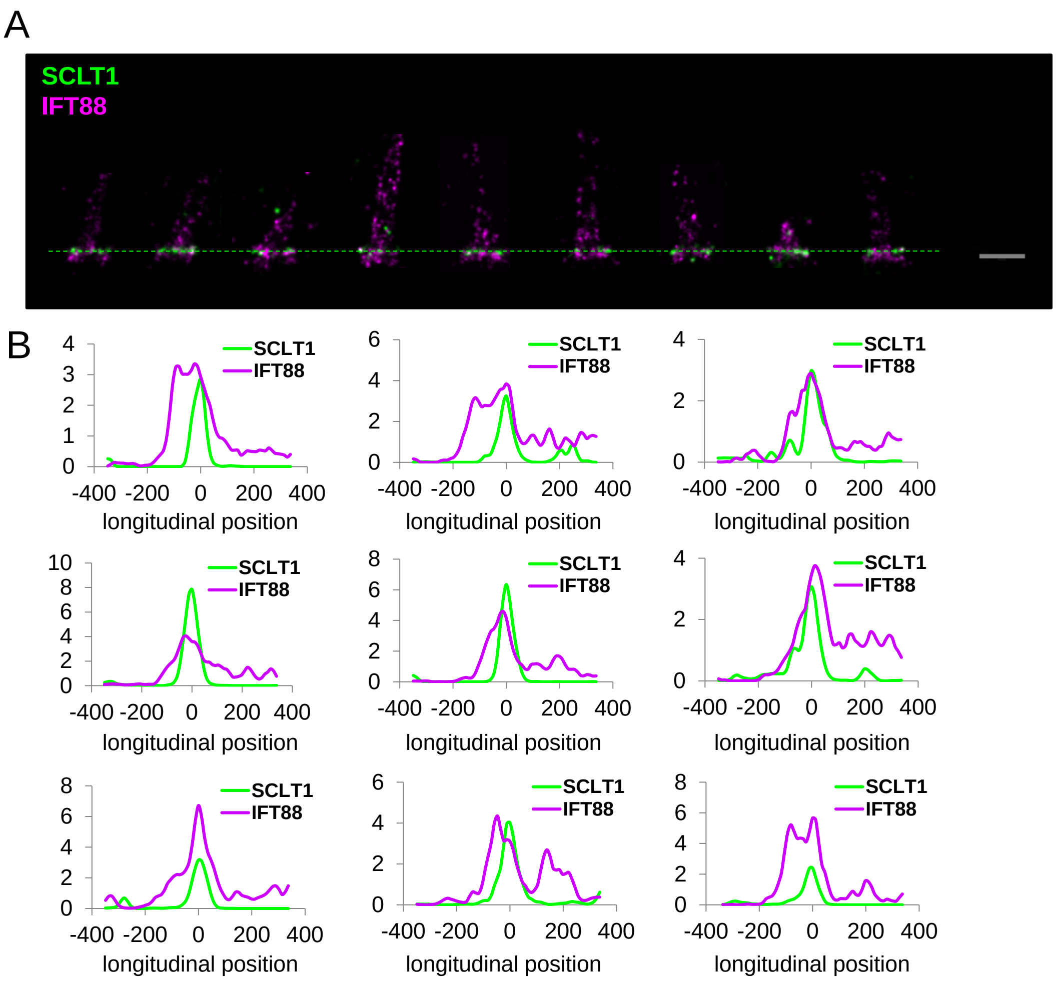

Supplementary Figure 12 Longitudinal distribution of IFT88.

(A and B) Two-color dSTORM imaging of IFT88 and SCLT1 reveal that IFT88 proteins accumulate at the longitudinal position of SCLT1, with a broader distribution toward the distal side. Bar = 500 nm.

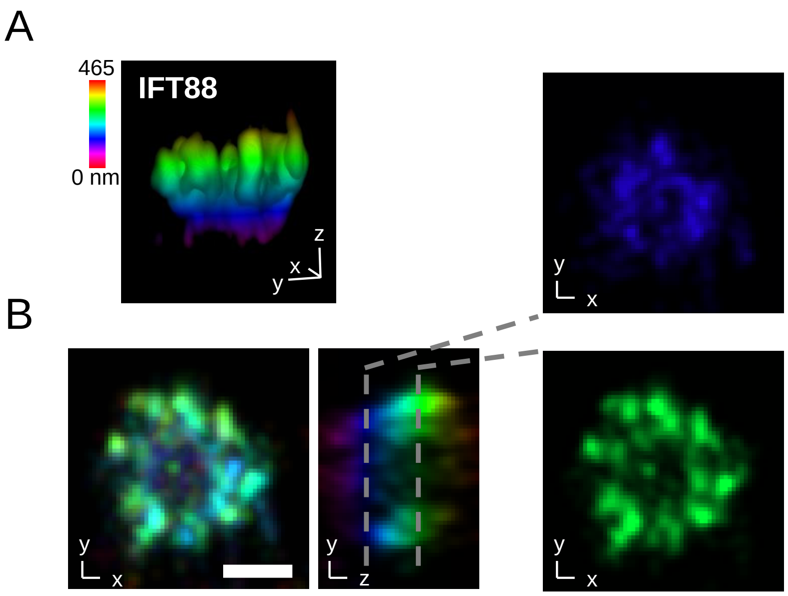

Supplementary Figure 13 3D superresolution imaging revealing the conical distribution of IFT88 at the DAP region.

(A) 3D dSTORM image showing the tapered distribution of IFT88 at the DAP region.

(B) XY and YZ view images of the results in (A) showing IFT88 radial patterns of different diameters extracted from the two different Z planes (right). Bar = 200 nm.

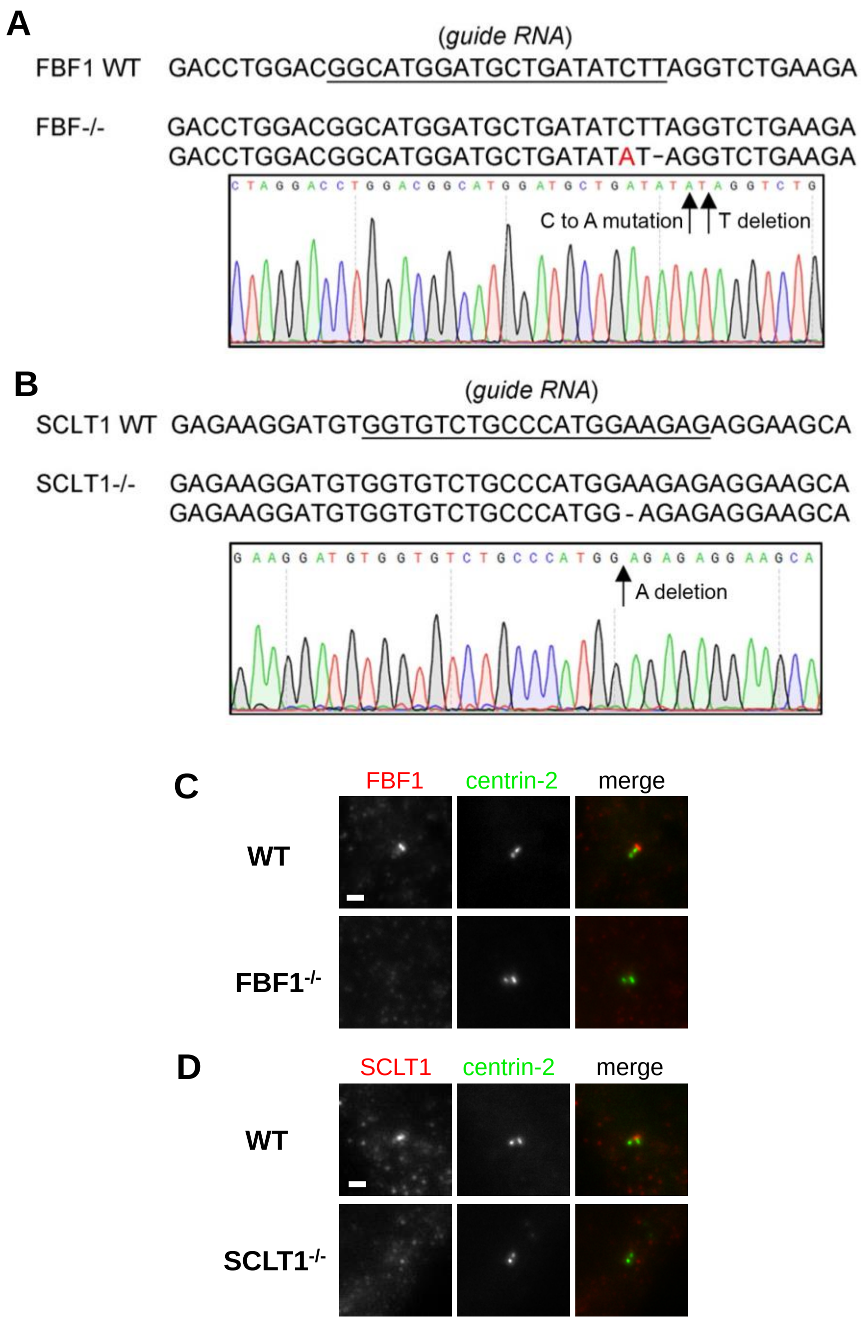

Supplementary Figure 14 Sequencing and immunostaining validation of CRISPR knockout *FBF1*$^{-/-}$ and *SCLT1*$^{-/-}$ cell lines established through clonal propagation from single cells.

(A) In the *FBF1*$^{-/-}$ cell line, a frameshift mutation in the coding region was found in each FBF1 allele, resulting in truncated products consisting of 88 amino acids. (B) In

the *SCLT1*$^{-/-}$ cell line, a frameshift mutation in the coding region was found in each SCLT1 allele, producing truncated products consisting of 282 amino acids. (C and D) Immunostaining of *FBF1*$^{-/-}$ and *SCLT1*$^{-/-}$ RPE-1 cells showing the effective knockout of the corresponding proteins compared with wild-type (WT) RPE1 cells. Bars = 1 μm.

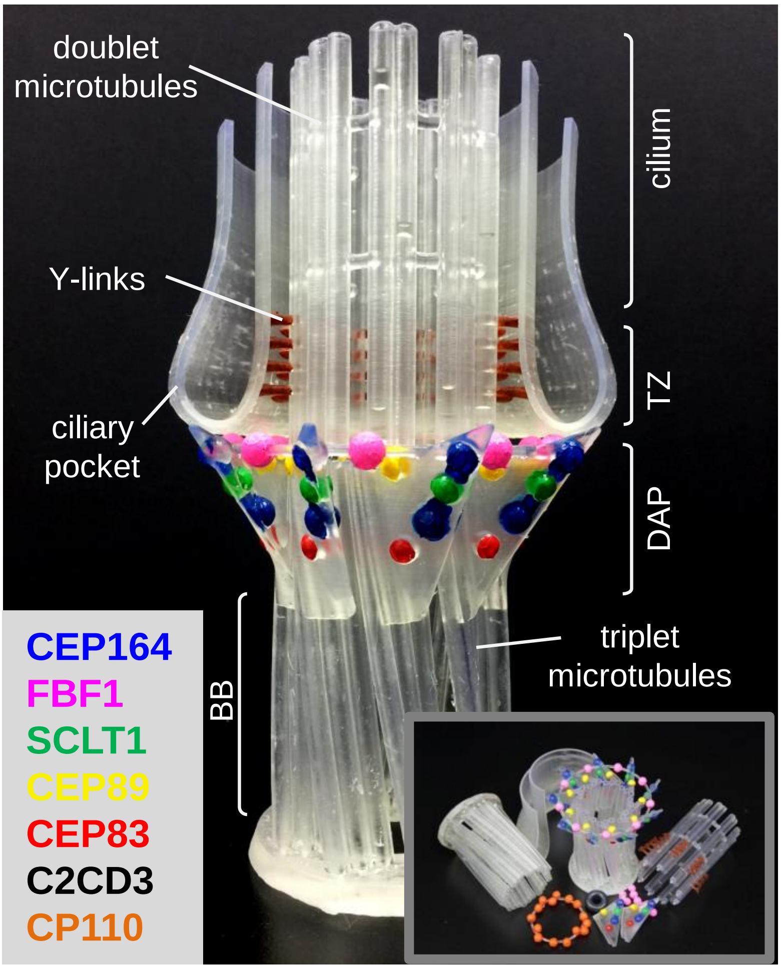

Supplementary Figure 15 A 150,000:1 3D printing model of the distal appendage (DAP) region, the transition zone (TZ), the basal body (BB), the axoneme, the ciliary membrane, and the ciliary pocket.

Distinct spatial arrangements of the DAP-associated proteins are marked in different colors. CEP83, CEP89, SCLT1, and CEP164 are mapped to different parts of DABs, whereas FBF1 localizes to the distal end of the DAM. Disassembled individual components are shown in the lower-right panel.

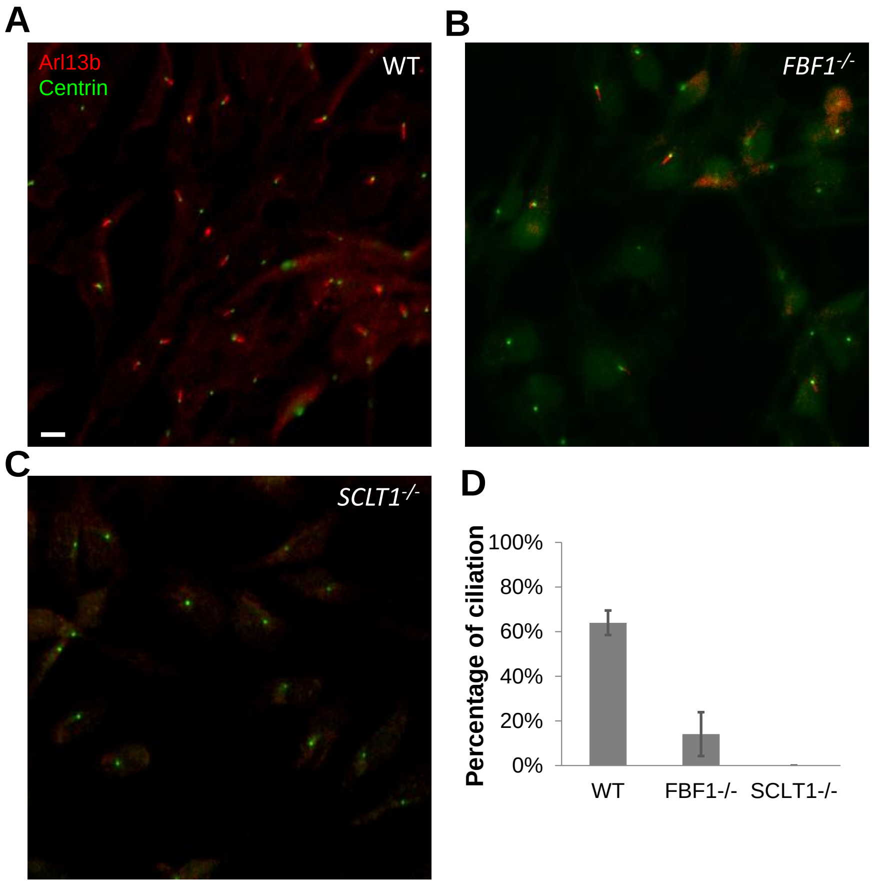

Supplementary Figure 16 Ciliogenesis in *FBF1*$^{-/-}$ and *SCLT1*$^{-/-}$ knockout cell lines. (A–C) Widefield imaging showing that ciliogenesis occurred in a small percentage of *FBF1*$^{-/-}$ cells, whereas no ciliation was observed in *SCLT1*$^{-/-}$ cells. Bar = 10 μm. (D) Formation of cilia was disrupted by ~85% in FBF1-deleted cells. Statistical analysis of three independent experiments was performed on 464, 572, and 246 WT, *FBF1*$^{-/-}$, and *SCLT1*$^{-/-}$ cells, respectively. Data are presented as mean ± s.d.

Supplementary Table 1 Superresolved radial and longitudinal locations of distal appendage-associated proteins

Proteins	Diameter (mean ± s.d., nm)		Longitudinal position relative to CEP83 (mean ± s.d., nm)	
**Axoneme**	~170	-	-	-
**C2CD3**	76 ± 13	(7 MCs)	-19 ± 9	(10 MCs)
**CEP290**	179 ± 35	(7 MCs)	-	-
**CEP162**	185 ± 29	(9 MCs)	-	-
**CEP97**	172 ± 35	(6 MCs)	71 ± 10	(10 MCs)
**CP110**	196 ± 24	(10 MCs)	75 ± 11	(10 MCs)
**Cby1**	266 ± 40; 447 ± 56	(149 puncta, 9 MCs)	49 ± 17; 3.5 ± 11	(9 MCs)
**ODF2**	278 ± 27	(6 MCs)	-19 ± 27; -145 ± 31	(50 puncta)
**CEP83**	318 ± 20	(13 MCs)	0	-
**CEP89**	352 ± 45	(8 MCs)	86 ± 7; -47 ± 28	(12 MCs)
**SCLT1**	419 ± 36	(8 MCs)	59 ± 8	(6 MCs)
**FBF1**	429 ± 23	(12 MCs)	89 ± 11	(10 MCs)
**CEP164**	381 ± 70; 475 ± 39	(15 MCs, 241 puncta)	85 ± 9; 24 ± 7	(6 MCs)

MC = mother centriole

Supplementary Table 2 Results of angular position analysis

Proteins	Difference in angular position of the first adjacent puncta between paired proteins (mean ± s.d., degrees)	Analyzed region (degrees)	Number of measured points
CEP83-SCLT1	39.03 ± 8.02°	[20, 60]	315 (4 MCs)
CEP89-CEP164	38.55 ± 9.10°	[20, 60]	454 (7 MCs)
CEP164-SCLT1	40.57 ± 8.87°	[20, 60]	395 (5 MCs)
FBF1-CEP164	19.17 ± 7.66°	[0, 40]	754 (7 MCs)
FBF1-SCLT1	19.44 ± 9.19°	[0, 40]	561 (7 MCs)

Supplementary Table 3 Primary antibodies used in this study

Name of antibody	Host	Company	Cat	Dilution	Immunogen (sequence)	Reference
CEP164	rabbit IgG	Novus Biologicals, Littleton, CO, USA	45330002	1/2000	human CEP164 middle region	Maskey, Marlin et al. 2015
CEP164	goat IgG	Santa Cruz Biotechnology, Dallas, TX, USA	sc-240226	1/200	human CEP164 N-terminus	Kong, Farmer et al. 2014
FBF1	rabbit IgG	Proteintech, Rosemont, IL, USA	11531-1-AP	1/200	FBF1 N-terminus (20-347aa)	Wei, Xu et al. 2013
SCLT1	rat IgG	Tanos et al. *Genes & development* (2013)	-	1/250	-	Tanos, Yang et al. 2013
CEP89	rat IgG	Tanos et al. *Genes & development* (2013)	-	1/500	-	Tanos, Yang et al. 2013
CEP83	rabbit IgG	Sigma-Aldrich, St. Louis, MO, USA	HPA038161	1/200	human CEP83 (amino acids 578-677)	Kong, Farmer et al. 2014
Cby1	mouse IgG	Santa Cruz Biotechnology	sc-101551	1/200	human Chibby (amino acids 1-63)	Zou, Tian et al. 2013
CP110	rabbit IgG	Proteintech	12780-1-AP	1/500	Recombinant human CP110 protein (Proteintech, Ag3489)	Cao, Shen et al. 2012
CEP97	rabbit IgG	Proteintech	22050-1-AP	1/150	CEP97 Fusion Protein (Proteintech, Ag16995)	Prosser and Morrison 2015
CEP162	rat IgG	Wang et al., *Nature cell biology* (2013)	-	1/1000	-	Wang, Tay et al. 2013
CEP290	rabbit IgG	Abcam, Cambridge, UK	ab84870	1/500	human CEP290 (a region between residues 2429 and 2479)	Silva, Betleja et al. 2016
C2CD3	rabbit IgG	Sigma-Aldrich	HPA038552	1/1000	human C2CD3 (amino acids 2122-2205)	Balestra, Strnad et al. 2013
IFT88	rabbit IgG	Proteintech	13967-1-AP	1/200	human IFT88 C-term (last 302 a.a.)	Kodani, Salome Sirerol-Piquer et al. 2013
TTBK2	rabbit IgG	Sigma-Aldrich	HPA018113	1/500	human TTBK2 (amino acids 819-921)	Cajanek and Nigg 2014
ODF2	rabbit IgG	Sigma-Aldrich	HPA001874	1/200	human ODF2 (amino acids 39-200)	Asante, Maccarthy-Morrogh et al. 2013
EHD1	rabbit IgG	Abcam	ab109311	1/200	Synthetic peptide corresponding to residues in Human EHD1	Cypher, Bielecki et al. 2016
Arl3b	mouse IgG	Abcam	ab136648	1/500	Fusion protein amino acids 208-427 (C-terminus) of mouse Arl13b.	Cypher, Bielecki et al. 2016
Centrin	mouse IgG	Millipore, Billerica, MA, USA	04-1624	1/400	C-terminus of Chlamydomonas Centrin	Seo, Jang et al. 2015
Polyglutamylated tubulin (GT335)	mouse IgG	AdipoGen, San Diego, CA, USA	AG-20B-0020-C100	1/500	Octapeptide EGEGE*EEG, modified by the addition of two glutamyl units onto the fifth E (indicated by an asterisk)	Hilbert, Noga et al. 2016

Supplementary Reference

1. Ishikawa, H., Kubo, A., Tsukita, S. & Tsukita, S. Odf2-deficient mother centrioles lack distal/subdistal appendages and the ability to generate primary cilia. *Nat. Cell Biol.* **7**, 517-524 (2005).
