# Supplementary material for: Super-resolution architecture of mammalian centriole distal appendages reveals distinct blade and matrix functional components
Source: Nat Commun. 2018 May 22;9:2023. doi: 10.1038/s41467-018-04469-1 (PMC5964178; doi:10.1038/s41467-018-04469-1)
Supplement: Supplementary file 3 — Description of Additional Supplementary Files [file 41467_2018_4469_MOESM3_ESM.pdf]

Description of Additional Supplementary Files

File Name: Supplementary Movie 1
Description: 3D dSTORM imaging showing the peripheral distribution of EHD1 surrounding the primary cilium.

File Name: Supplementary Movie 2
Description: 3D two-color dSTORM imaging of IFT88 and SCLT1 showing the localization of IFT88 at the gaps between neighboring SCLT1 regions.

File Name: Supplementary Movie 3
Description: 3D view of the centriole-cilium model including proteins localized at the distal appendage matrix (DAM) and the distal appendage blades (DABs).
